# Convergence of plasmid architectures drives emergence of multi-drug resistance in a clonally diverse Escherichia coli population from a veterinary clinical care setting

**DOI:** 10.1016/j.vetmic.2017.09.016

**Published:** 2017-11

**Authors:** Sam Wagner, Nadejda Lupolova, David L. Gally, Sally A. Argyle

**Affiliations:** The Royal (Dick) School of Veterinary Studies and The Roslin Institute, The University of Edinburgh, Roslin, Midlothian, United Kingdom

**Keywords:** IncI1, IncF, Unrinary tract infection, Dog, Beta-lactamase, *bla*_CMY-2_

## Abstract

The purpose of this study was to determine the plasmid architecture and context of resistance genes in multi-drug resistant (MDR) Escherichia coli strains isolated from urinary tract infections in dogs. Illumina and single-molecule real-time (SMRT) sequencing were applied to assemble the complete genomes of *E. coli* strains associated with clinical urinary tract infections, which were either phenotypically MDR or drug susceptible. This revealed that multiple distinct families of plasmids were associated with building an MDR phenotype. Plasmid-mediated AmpC (CMY-2) beta-lactamase resistance was associated with a clonal group of IncI1 plasmids that has remained stable in isolates collected up to a decade apart. Other plasmids, in particular those with an IncF replicon type, contained other resistance gene markers, so that the emergence of these MDR strains was driven by the accumulation of multiple plasmids, up to 5 replicons in specific cases. This study indicates that vulnerable patients, often with complex clinical histories provide a setting leading to the emergence of MDR *E. coli* strains in clonally distinct commensal backgrounds. While it is known that horizontally-transferred resistance supplements uropathogenic strains of E. coli such as ST131, our study demonstrates that the selection of an MDR phenotype in commensal E. coli strains can result in opportunistic infections in vulnerable patient populations. These strains provide a reservoir for the onward transfer of resistance alleles into more typically pathogenic strains and provide opportunities for the coalition of resistance and virulence determinants on plasmids as evidenced by the IncF replicons characterised in this study.

## Highlights

• Multi-drug resistant *E. coli* associated with urinary tract infections in dogs have a commensal strain background.
• Beta-lactam resistance is associated with *bla*_CMY-2_ located exclusively on a highly clonal IncI1 plasmid.
• IncI1 plasmids carried no other identifiable resistance genes.
• Isolates in some cases carried up to 5 plasmids, responsible for carriage of the additional resistances.

## 1 Introduction

*E. coli* is an important commensal organism and a significant pathogen. It is associated with intestinal and extra-intestinal infections and is a leading cause of urinary tract infections (UTIs) and bacteraemia leading to sepsis (Pitout, 2010).

Beta-lactam antimicrobials are widely used in both human and animal medicine. Due to their spectrum of activity, pharmacokinetic characteristics and good safety profile, members of the group are a good choice in the treatment of UTIs associated with *E. coli*. However, there is increasing resistance to these antimicrobials, mediated by the ability of Enterobacteriaceae such as *E. coli*, to produce plasmid mediated AmpC (pAmpC) and extended spectrum beta-lactamase enzymes (ESBLs). This imparts resistance to most of the Beta-lactam antimicrobials, including the later generation cephalosporins. In addition, many are also resistant to other antimicrobial classes rendering them multi-drug resistant (MDR), significantly increasing morbidity and mortality (Harris, 2015). The prevalence of pAmpC and ESBL resistance is increasing in both hospital and community acquired infections (Nakai et al., 2016). Less information is available for companion animals, but studies that have evaluated this report a resistance epidemiology similar to that observed in human clinical care (Dierikx et al., 2012, Gibson et al., 2010, Murphy et al., 2010, Tamang et al., 2012)

Successful dissemination of resistance within MDR *E. coli* is attributable to the fact that they are mostly located on horizontally transferable elements (HTEs) such as plasmids and transposable elements. HTEs are often promiscuous with a diverse bacterial host range, conferring resistance not just within but also between bacterial species, generating a large potential resistance reservoir (Bortolaia et al., 2011). It is important to determine the genetic context of resistance alleles to understand co-inheritance of traits that may drive selection and the potential of resistances to coalesce in single isolate backgrounds.

As sequencing technologies have advanced, an increasing number of resistant E. coli strains, have been sequenced. Initially, much of the sequence comparison relied upon read mapping and short read de novo assembly. These methods often fail to accurately resolve complete chromosomes and other replicons due to multiple repeat regions which are often present in mobile genetic elements. As a consequence, there is a relative lack of availability of high quality sequence data relating to complete bacterial plasmids encoding antibiotic resistance. The advent of long read sequencing technologies is now leading to increased representation of these sequences in public databases.

The aim of this study was to characterise MDR *E. coli* associated with urinary tract infections in dogs focusing on the use of Illumina sequencing combined with Pacific Biosciences single molecule real time (SMRT) sequencing to elucidate the plasmid repertoires that have been assembled to generate MDR isolates.

## 2 Materials and methods

### 2.1 Bacterial strains

All isolates (16 MDR and 14 antibiotic susceptible (AS)) were previously characterized in terms of bacterial identification, phylogenetic group, plasmid replicon type, MDR phenotype and ST type (Wagner et al., 2014).

### 2.2 Illumina sequencing

*E. coli* isolates were cultured overnight at 37 °C, 170 rpm, in lysogeny broth (LB). DNA was extracted using Qiagen DNeasy extraction kit (Qiagen U.K.). DNA was isolated from 1 ml of bacterial culture, according to the manufacturer's specifications. Following integrity assessment on agarose gels and quantification/quality determination by spectrophotometry (including absorbance 260:280 nm), Nextera XT libraries were prepared by Edinburgh Genomics. Paired-end Illumina sequencing was performed using an Illumina Hi-Seq 2500 sequencer with read lengths of 100 bp to achieve an average depth of 60×.

### 2.3 SMRT sequencing

SMRT sequencing was performed using the PacBio platform (Pacific Biosciences). *E. coli* were inoculated into 10 ml LB and incubated overnight at 37 °C, 170 rpm. DNA was extracted using the Qiagen 100/G extraction kit, according to the manufacturer's instructions. Extracted DNA was prepared for sequencing using AMPure Beads (Beckman Coulter), with a target insert size range of 10 kb or greater. DNA was sheared using a g-TUBE™ (Covaris). Sheared DNA was purified and concentrated using AMPure beads. Samples were eluted using PacBio Elution Buffer, and single stranded fragments were removed with ExoVII DNA. Fragments were then repaired using the PacBio DNA Damage and Repair Ends buffers, as per the protocol. Processed DNA was further purified using AMPure beads; then blunt ended sequence adapters were ligated, and ExoIII and ExoVII restriction enzymes used to remove any failed ligation products. Successfully ligated sequence fragments were concentrated using three successive AMPure bead purification steps. Final DNA concentration for all of the samples exceeded 5 μg. DNA was sequenced on the PacBio RSII sequencing platform.

### 2.4 Sequence assembly and annotation

Illumina sequence reads were filtered using Sickle (Joshi and Fass, 2011) with a minimum read quality score of 30, and a read length of 50 bp. Reads were re-shuffled with shuffleSequences fastq.pl script (Zerbino, 2010). De novo assembly used both paired reads and singletons. Illumina sequence reads were assembled with the VelvetOptimiser script, from Velvet 1.2.08 (Zerbino and Birney, 2008) using a kmer size range of 47 to 67.

SMRT analysis was used to generate a fastq file from the PacBio reads and error-corrected reads were adjusted using PBcR with self-correction (Koren et al., 2013). Then the longest 20× coverage reads were assembled with Celera Assembler 8.1 and polished using Quiver (Chin et al., 2013). Annotated genomes (Do-It-Yourself Annotator (DIYA) (Stewart et al., 2009)) were imported into Geneious (Biomatters LTD., Auckland, New Zealand) (Kearse et al., 2012) and duplicated sequence removed from the 5′ and 3′ ends to generate the circularized chromosomes/plasmids. Origin of replication was approximated using Ori-Finder (Luo et al., 2014) and the chromosome reoriented using the origin as base 1.

### 2.5 Core genome analysis

To construct core SNPs trees short reads were aligned with BWA http://www.ncbi.nlm.nih.gov/pubmed/19451168 (Li and Durbin, 2009) to a reference *E. coli* O157:H7 Sakai strain (RefSeq assembly accession: GCF\_000008865). Consensus sequence for each alignment of 5,590,092 bp was produced using the majority rule and then used to detect and remove recombination regions with Gubbins http://nar.oxfordjournals.org/content/early/2014/11/20/nar.gku1196.short (Croucher et al., 2015).

Resulting sequences were used to construct a Maximum Likelihood (ML) tree with RAxML http://www.ncbi.nlm.nih.gov/pubmed/24451623 (Stamatakis, 2014) under a GAMMA model of heterogeneity with 500 bootstrap replicates.

### 2.6 Plasmid sequence comparison

CCTViewer (Grant et al., 2012) was used to visualise SMRT sequenced plasmids, using 1428 p96 as a reference sequence for the IncI1 plasmids (Accession no CP023370) and 144 p134 (Accession no CP023363) as the reference for the IncF plasmid (Table 2). ProgressiveMauve (Darling et al., 2010) alignment was performed using default parameters. ResFinder (Zankari et al., 2012) was used to confirm annotated antimicrobial resistance markers. BlastKOALA (Kanehisa et al., 2016) was used to identify putative virulence factor sequences on the plasmid.

**Table 1:** Summary of the *E. coli* reference strains used in this study.

*E. coli* reference strain identity	Accession number	Description
UTI89	CP000243.1	Uropathogenic
CFT073	AE014075.1	Uropathogenic
536	CP000247.1	Uropathogenic
ABU83972	CP001671	Asymptomatic bacteruria
JJ1886	CP006784	Multi-drug resistant
NA114	CP002797	Multi-drug resistant uropathogenic
MG1655	NC_000913	Non pathogenic
UMNK88	NC_017641.1	Porcine enterotoxigenic
Sakai 0157:H7	BA000007.2	Human enterohaemorrhagic
APEC O78	CP004009.1	Avian pathogenic

**Table 2:** Plasmid descriptions from isolates following Illumina and SMRT sequencing.

Name^*^	CDS	Plasmid MLST	Resistance genes	Virulence genes
127 p123	140	IncFII: 22 FIA: 6 FIB: 69	*aadA5*, *aph*(*3′)-1a*, *strB*, *strA*, *catA1*, *sul1, sul2*, *dfrA17*	–
127 p95	105	IncI1:ST2 (CC2)	*bla*_CMY-2_	–
127 p91	100	nt	–	–
127 p43	51	nt	*bla*_TEM-33_, *dfrA1*	*hha, virD, virB*
127p39	48	nt	–	*fimD, hha, virD, virB*
1223 p147	150	IncFII:18	–	*iss, iroN, mchF*
1223 p87	97	IncI1:ST23 (CC2)	*bla*_CMY-2_	*dot*
1283 p109	128	IncFII:31	*tet*(A)	*TC·FEV.OM3, sit*
1283 p92	101	IncI1: ST2 (CC2)	*bla*_CMY-2_	*dot*
1283 p31	41	nt	–	*hha, virD, virB*
1283 p7	9	nt	*strA, strB, sul2*, *dfrA14*	–
1283 p3	3	nt	–	–
1428 p111	130	IncFII:19 FIB:27	–	*ompT, TC·FEV.OM2, fes, iroC*
1428 p96	113	IncI1:ST43 (CC-)	*bla*_CMY-2_	*dot*
1428 p66	108	IncFII:4	*tet*(B)	–
1428 p48	65	nt	–	–
144 p134	142	IncFII:2 FIB:1	*strA, strB*, *bla*_TEM-1b_, *sul2, dfrA5*	*iroC, sit, iuc, mer*
144 p92	105	IncI1:ST2 (CC2)	*bla*_CMY-2_	–
317 p100	105	IncFII:43	–	*ABC,LPT.P, TC·OOP, raxB*
746 p95	106	IncI1:ST2 (CC2)	*bla*_CMY-2_	*dot*
746 p72	116	IncFII:2 §	*–*	*–*
746 p62	102	IncFII:2 §	*–*	*–*
1943 p85	90	IncI1:ST55 (CC-)	*bla*_CMY-2_	*dot*
1943 p80	86	IncFIB:1	*–*	*sit, iuc*
1943 p54	59	nt	**bla*_TEM-1a_, *cat*B3, *dfr*A1*	*hha, virD, virB*

^*^Each plasmid is referred to by the isolate ID followed by the letter p and then a number which represents the size of the plasmid in KB. nt = non typable, CC = clonal complex, − = non identified, § = putative split contig, CDS = coding sequence, ST = sequence type.

### 2.7 Analysis of plasmid core and pan genomes

Plasmid replicon and clonal complex types were determined using the pubMLST database (Jolley and Maiden, 2010). Core sequence homology between the plasmid sequences was detected with GET_HOMOLOGUES (Altschul et al., 1997) using bi-directional best-hits (BDBHs) and orthoMCL algorithms. Protein clusters were aligned with Muscle (Edgar, 2004). Amino acid alignments were then translated back into nucleotide sequences using the PAL2NAL (Suyama et al., 2006), concatenated and transformed into phyml format with catfasta2phyml/catfasta2phyml.pl script to use for RaXML phylogenetic estimation under a GTR model and 100 bootstrap replicates (Stamatakis, 2014). GET_HOMOLOGUES was also used for the pan genome analysis, using the PARS program from the PHYLIP package (Contreras-Moreira and Vinuesa, 2013).

## 3 Results

### 3.1 Comparative analysis of E. coli strains

**Fig. 1:**
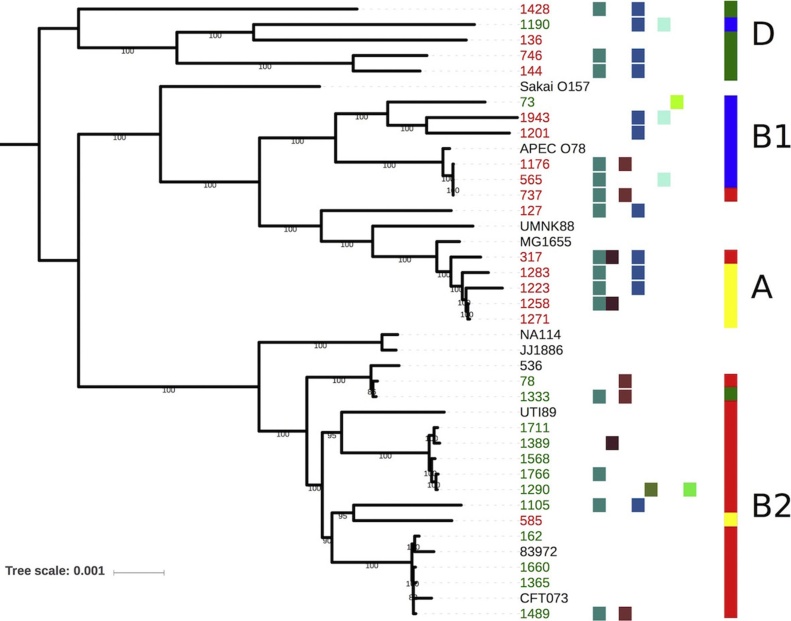
Maximum-parsimony tree of *E. coli* chromosomal sequence alignment. Isolates from this study are listed in red (MDR) or green (AS). Ref-Seq sequences are listed in black. Plasmid replicon types are designated from left to right by the colour coded squares: FII  FIA  FIB  I1  I2  B/O  ×1  R . Phylogroup designations are colour coded in the right hand column as follows, A = yellow, B1 = blue, B2 = red and D = green. (For interpretation of the references to colour in this figure legend, the reader is referred to the web version of this article.).

Core SNP-based phylogenetic analysis of the 30 clinical UTI associated isolates (16 MDR and 14 AS) plus 10 reference strains (Table 1) was carried out, and identified at least two distinct clusters that mostly correlates well with the isolates’ MDR status (Fig. 1). Isolates were ancestrally diverse, however there was a trend for susceptible isolates to be more closely associated to human UPEC *E. coli* reference sequences and MDR isolates clustered together with commensal *E. coli* reference sequences. Whole genome sequencing (WGS) analysis of the MDR strains confirmed previous standard phylotyping results regarding their diverse commensal backgrounds. Isolates that possess the IncI1/IncF plasmid genotype were well dispersed throughout the tree. Tree clades were largely independent with respect to date of isolation.

### 3.2 Sequencing and plasmid carriage

**Fig. 2:**
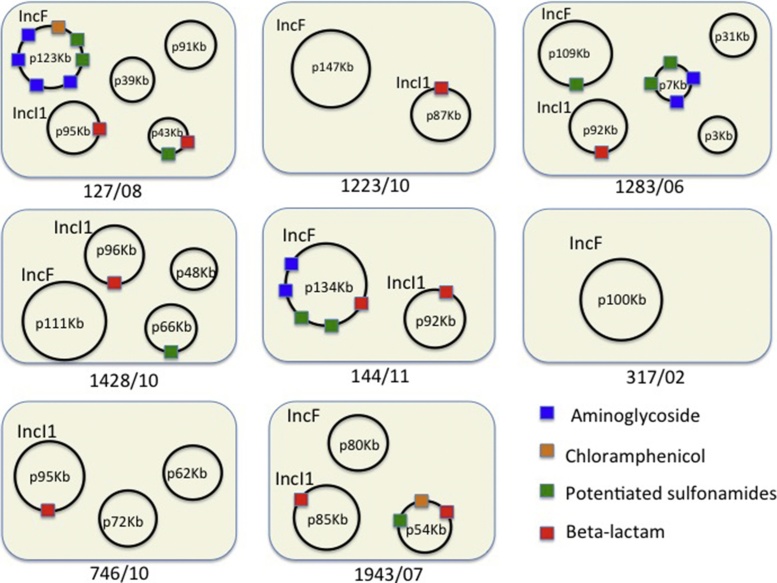
Graphical representation of the plasmid carriage of the *E. coli* isolates sequenced in this study. Boxes represent each isolate referred to by their isolate ID. Depicted sequences are not to scale. Plasmid sequences are identified and distinguished by their sequence length. Plasmid encoded resistance, where detected by BLAST search, is indicated by colour coding on the plasmids. Plasmid incompatibility type, where typable, is indicated (either IncF or IncI1). Chromosomal sequences are not depicted.

Plasmid sequences could not be assembled using Illumina short read sequence data. Therefore, 8 MDR isolates (127, 1223, 1283, 1428, 144, 317, 746 and 1943 (Supplementary Table S1)) were sequenced by SMRT, these were selected to examine the IncI1 replicon context of the CMY-2 (AmpC) beta-lactamase. Two sensitive isolates (1190 and 1105, (Supplementary Table S2)) but carrying an IncI1 replicon were also sequenced by SMRT. The plasmid combinations present in the MDR strains are described in Table 2 and depicted in Fig. 2 along with their contribution to MDR as defined by carriage of specific resistance alleles.

While 7/8 MDR strains had the anticipated replicons, one isolate (317) only carried an IncF plasmid, despite PCR and Illumina sequence data indicating the presence of IncF and IncI1 replicons. It is possible that the plasmid was lost during the experimental process. One susceptible isolate, 1105, was PCR positive for the IncI1 genotype, but this replicon could not be assembled from the SMRT sequence data.

### 3.3 IncI1 comparative analysis

**Fig. 3:**
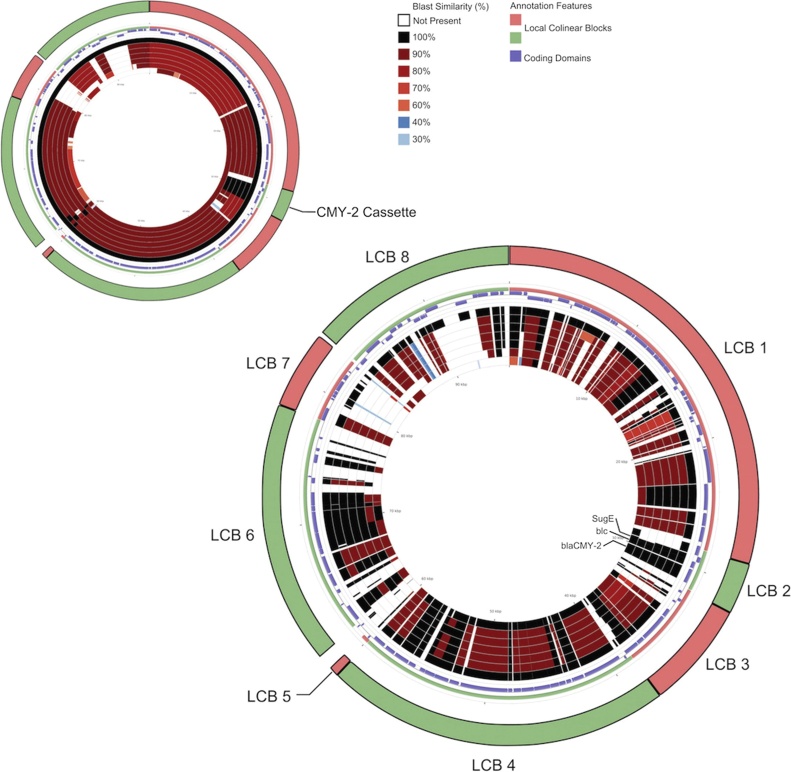
Core sequence alignment of IncI1 replicon type plasmid sequences, using 1428 p96 as the index sequence. Comparisons were performed using a BLAST Atlas search using the CCT comparison tool. Both the whole nucleotide sequence (smaller ring) and coding domain sequence specific (larger ring) BLAST comparisons were carried out. ProgressiveMauve was also used to compare the plasmids. Local co-linear blocks, detected by progressiveMauve have been annotated onto the BLAST comparisons.

In the MDR isolates, the IncI1 plasmid sequence sizes ranged from 85 to 96 kb, with between 106 and 113 coding domain sequences; many of which still had no ascribed function. All but two of the IncI1 plasmids belonged to the same clonal complex (CC-2), determined in silico, based on the presence and sequence similarity of *repI1*, *ardA*, *trbA*, *sogS*, and *pilL* to previously published IncI1 pMLST profiles. As anticipated, the IncI1 plasmids associated exclusively with the CMY-2 encoded pAmpC beta-lactamase resistance gene; this was located within a resistance cassette associated with a mobile element (Fig. 2, Fig. 3).

Significant periods of time separate isolation of the sequenced strains; the first collected in 2001, the most recent 2011. This makes the comparison of the IncI1 replicons unique as it provides insight into the evolution of this resistance-encoding replicon in our local context over this time period.

BLAST Atlas search using the CCT comparison tool for analysis of the Incl1 replicons show greater than 90% sequence similarity for most of the sequences. Multiple collinear blocks, with high synteny and sequence similarity and limited large-scale rearrangements were identified using progressive Mauve (Fig. 3).

**Fig. 4:**
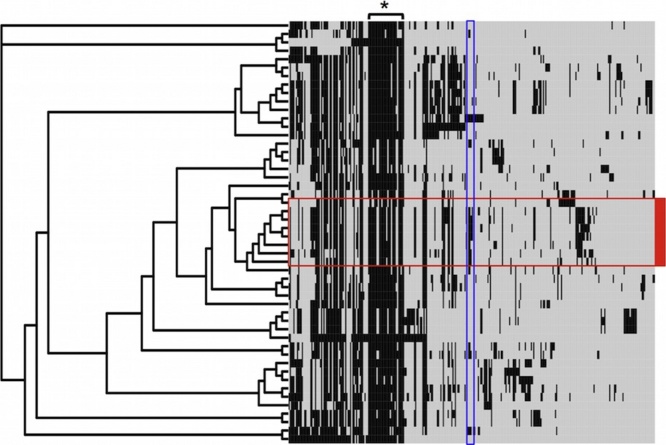
Maximum-parsimony analysis using detected presence or absence of homologous gene clusters from the pan genome of PacBio IncI1 replicon sequences, and comparator IncI1 sequences obtained from the NCBI nucleotide sequence database. Plasmid sequences from this study lie within the red box. Core genome genes (used for maximum-likelihood analysis) are indicated by *. The CMY-2 resistance cassette, common to the IncI1 plasmid sequences isolated in this study, is highlighted by the blue box. (For interpretation of the references to colour in this figure legend, the reader is referred to the web version of this article.)

IncI1 plasmids were compared against 42 plasmid sequences obtained through GenBank, which were identified by BLAST as being similar (Supplementary Fig. 1). Pan-genomic analysis detected 305 homologous gene clusters across all sequences. Phylogenetic estimations using homologous cluster presence or absence indicated a close phylogenetic relationship between all IncI1 plasmids sequenced in this study including the IncI1 plasmid from the susceptible *E. coli* isolate in this study (1190/01 Accession no CP023387). The resistance cassette conferring CMY-2 mediated pAmpC resistance although not exclusive to the canine resistance plasmids, was not detected in many other IncI1 sequences available on databases. Ten of the homologous gene clusters: *traL*, *traM*, *nikB*, *traO*, *traJ*, *traF*, *traE*, *traT*, *traI*, and *traX*; all relating to plasmid transfer and replication were core to all the IncI1 plasmid sequences (Fig. 4). These were extracted from the pan-genomic analysis and aligned for maximum likelihood core genome phylogenetic estimation (Supplementary Fig. 1). Despite only weak bootstrap support overall, the phylogenetic tree is congruent with the predicted in silico MLST plasmid groups. The IncI1 plasmids included in the analysis were from different *Salmonella* serovars and *E. coli* isolated from different animal hosts suggesting that interspecies transmission of the IncI1 plasmids occurs. However, IncI1 sequences associated with bacteria isolated from humans were largely absent, despite the over representation of human isolates in databases in general. The majority of canine sequences form a sub-cluster, with chicken- and porcine-associated plasmid sequences being the most similar. Of note, CP009566 and KF434766 are the only two reference IncI1 plasmids associated with canine clinical infections; isolated in *Salmonella enterica* serovar Newport in Arizona in 2015 (Cao et al., 2015) and *E. coli* in Denmark in 2008 (Hansen et al., 2016) respectively. These were closely related to the canine *E. coli* plasmids sequenced in this study even though their host bacterial strains were obtained from different geographical areas and at different times (Supplementary Fig. 1).

### 3.4 IncF comparative analysis

The IncF plasmids were more heterogeneous in size (100–147 kb), the number of coding regions (105–150), and the number and classifications of various resistance markers. Aminoglycoside, chloramphenicol, potentiated-sulfonamide, and beta-lactam resistance markers (other than CMY-2) were associated with IncF or smaller untyped plasmids (Fig. 2).

**Fig. 5:**
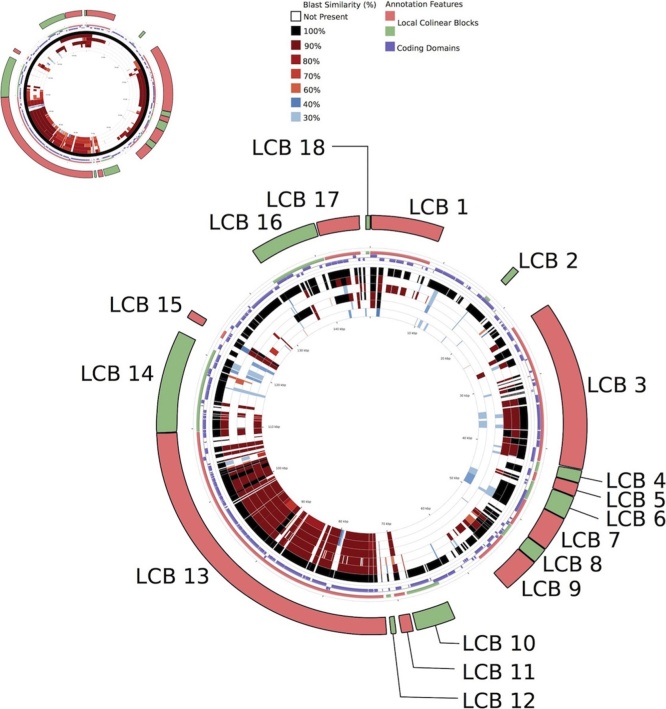
Core sequence alignment of IncFII replicon type plasmid sequences, using 1223 p147 as the index sequence. Comparisons were performed using a BLAST Atlas search using the CCT comparison tool. Both the whole nucleotide sequence (smaller ring) and coding domain sequence specific (larger ring) BLAST comparisons were carried out. ProgressiveMauve was also used to compare the plasmids. Local co-linear blocks detected by progressiveMauve have been annotated onto the BLAST comparisons.

**Fig. 6:**
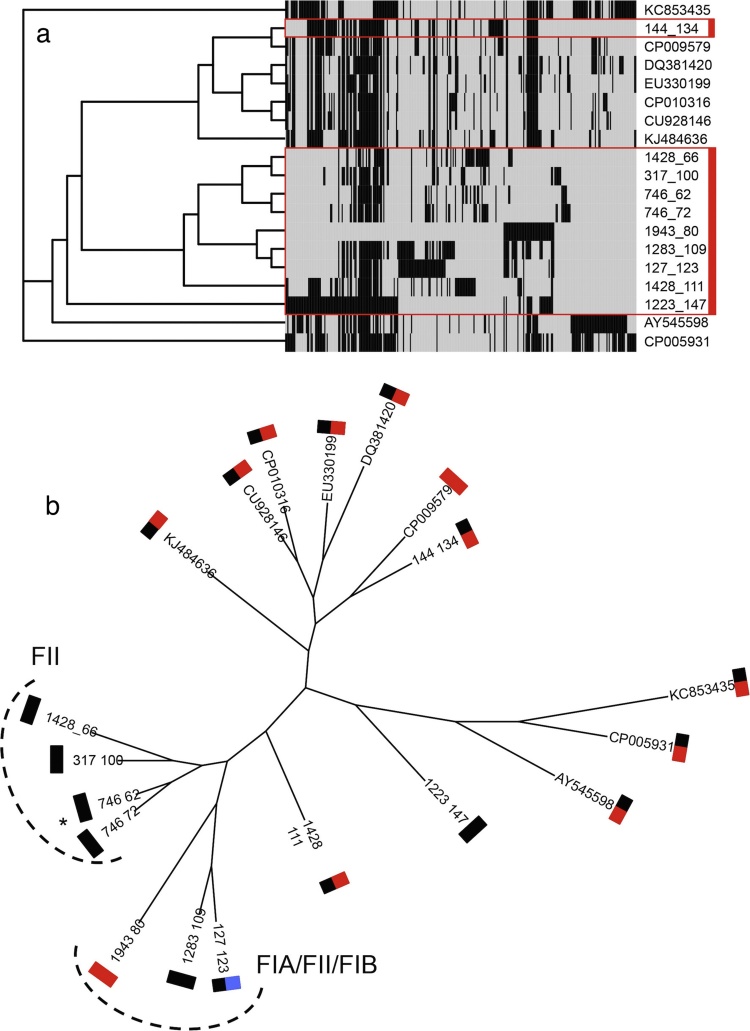
Maximum parsimony and maximum likelihood analysis for IncFII. a) Maximum parsimony analysis of pan-genomic homologous gene clusters for IncFII replicon sequences. Similar NCBI nucleotide sequences, with detectably diverse replicon types were included. Isolates sequenced in this study are indicated by the red boxes. b) Maximum-likelihood analysis of aligned core homologous sequences. Sequences have been annotated with their detected replicon type. FII  FIA  FIB . (For interpretation of the references to colour in this figure legend, the reader is referred to the web version of this article.)

Despite the greater variety and number of resistance markers shared amongst some of the IncFII plasmids, five contained no detectable resistance markers. Virulence genes, mostly associated with iron uptake, and genes associated with metabolism were detected on many of the IncF plasmids. In silico replicon typing indicates that many of these plasmids were of mixed lineage (Table 2). IncF plasmids showed less sequence similarity when compared by BLAST or by progressiveMauve sequence analysis. With one exception, all regions of local co-linearity had little synteny, or support across all the plasmid sequences (Fig. 5). GenBank BLAST searches identified 9 sequences similar to the IncFII/IncFIB plasmids. Pan-genomic maximum parsimony analysis indicated diverse content (Fig. 6a). No core genes could be identified for all the plasmid sequences, although a subset, excluding 1428 p66, 746 p62, 746 p72, and 1943 p80, could be compared using 8 homologous gene clusters; as with the IncI1 plasmids these genes were mostly associated with plasmid maintenance and replication, including *traA*, *traL*, *traE*, *traB* and *traX*. The different replicon sub-types are depicted in Fig. 6b, with FII/FIB the most commonly identified. A singular clade of plasmids, exclusively IncFII, showed greater sequence homology than the remaining plasmid sequences (Fig. 6a & b). Maximum likelihood phylogenetic analysis of IncFII, IncFIA, and IncFIB replicon types showed dispersal of all sequences of different replicon types throughout the tree (Fig. 6b). None of the PCR typed IncFII plasmids sequenced in this study were members of the same clade and 317 p100 was a significant outlier from the rest of the plasmid sequences.

### 3.5 Other plasmids

Numerous other extra-chromosomal contigs were detected from SMRT sequencing. IncI1, IncFII, IncFIA, and IncFIB, were the only detected incompatibility types, yet account for a fraction of plasmid sequences. The nature of the SMRT sequencing and the analysis means that these sequences are very unlikely to be part of the main chromosome or the plasmids with defined replicon-types. Whilst many of these do not contain identifiable resistance markers, some do. Many of these sequences do not have established replication and transfer machinery encoded on them, so their transfer capacity is currently unknown, but they may represent small replicons that can be co-inherited with other transferred plasmids or possibly by other methods such as transduction.

## 4 Discussion

MDR *E. coli* isolates are relatively rarely isolated from in and out-patient samples presented for testing at the University of Edinburgh’s small animal hospital. As previously described (Wagner et al., 2014) MDR isolates are often associated with animals with complex medical histories and understanding their emergence in the context of generally sensitive *E. coli* isolates (AS) provides an important opportunity to develop our understanding of MDR emergence in a clinical setting.

Combined use of Illumina and SMRT sequencing has allowed detailed examination of individual plasmids and strain background, as well as an overview of plasmid carriage in the context of individual isolates.

Phenotypic beta-lactamase resistance is attributed to pAmpC, encoded exclusively by the CMY-2 allele on IncI1 replicon plasmids, forming a notably closely related phylogenetic cluster, with high levels of homogeneity in the IncI1 plasmid sequence, despite the strains having been collected over a 10 year period. The CMY-2 allele was the only identifiable resistance gene present on this subset of IncI1 plasmids. This has been reported previously in CMY-2 carrying IncI1 plasmids isolated from canine, feline and human hosts (Bortolaia et al., 2014, Sidjabat et al., 2014). The majority of the MDR IncI1 plasmids share a closest common ancestor, using maximum-likelihood analysis of the core genome. This is either due to i) limited sequence divergence or ii) sequence convergence. Given the genetic distance between the susceptible IncI1 plasmids, the absence of any dominant *E. coli* clone associated with the IncI1 plasmids, the lack of any indication of potential bacterial host range of the plasmids (other than *E. coli*), and the discordance of sequence similarity with the chronological sequence of the *E. coli* isolates; it is difficult to identify which. The reliability of any estimation of rates of divergence between the different plasmids is questionable; the identification of a similar plasmid backbone in canine clinical isolates CP009566 and KF434766 from the USA and Scandinavia does suggest underlying core genome stability (Bogaerts et al., 2015, Bortolaia et al., 2014). CMY-2 has also been identified from faeces of healthy dogs in a number of geographical locations including the Netherlands, Mexico, France and Japan, (Baede et al., 2015, Haenni et al., 2014, Okubo et al., 2014, Rocha-Gracia et al., 2015) and where determined, the plasmid context is predominantly IncI1. This may indicate that the IncI1-CMY-2 is endemic to the commensal population especially in the dog. The common use of cephalosporins such as cephalexin in companion animal practice has been implicated in high prevalence carriage particularly in canine isolates (Haenni et al., 2014).

In comparison to the IncI1 plasmids, the IncF plasmids are more disparate, in core and pan-genome sequence content. This could be a consequence of experimental design, as the isolates for this study were collected based on their pAmpC production, the gene for which is present on the IncI1 plasmid. However other studies also provide evidence for the relative heterogeneity of the IncF plasmids (Villa et al., 2010), and relatively few sequences could be identified in the NCBI database, sharing nucleotide similarity with the IncF sequences in this study; with little conservation of resistance markers between one IncF plasmid and another. Another contrasting feature of the IncF plasmids, supported by other studies, was their greater compliment of resistance alleles and putative virulence-associated genes compared to the IncI1 plasmids. As most of the strains containing the IncI1/IncF plasmid combination specifically were associated with commensal phylogroups, we speculate that acquisition of IncF plasmids in combination with IncI1 drives emergence of normally commensal *E. coli* strains resulting in clinical disease in vulnerable patients, especially in the presence of antibiotic selection. All patients from which MDR isolates were identified could without exception fall into this categorisation having significant underlying disease, often multiple antibiotic treatments and some receiving immunosuppressant therapies (Wagner et al., 2014). The dispersion of resistance genes across different plasmids, in many of the isolates, suggests sequential acquisition perhaps whilst part of the normal flora of the gastrointestinal tract; as it is unlikely that the transfer of multiple plasmids would occur as a singular event. Commensal strains have previously been implicated as a significant component of the resistance reservoir (Marshall and Levy, 2011) whilst other studies have clearly identified the carriage of CMY-2 in more typical UPEC strains such as ST 131 (Dashti et al., 2014).

SMRT sequencing identified strains containing up to 5 plasmids contributing up to 300KB of additional genetic information. Much of the function of these coding regions is unknown Sequencing also revealed the presence of small non-typable plasmids of which we were previously unaware. It is assumed that the acquisition of so much extra-chromosomal DNA may be energetically costly to the bacterial host, and many of the plasmids may not be stably maintained in this combinatorial manner in these specific backgrounds without antibiotic selective pressure. The long-term stability of these plasmids within their host bacterial genomes is currently unknown, but investigation of this in future work would be of significant value.

The MDR *E. coli* strains characterised in this study were isolated from clinical cases with significant underlying disease, which had received often multiple courses of antimicrobial chemotherapy. This represents a model of the genetic and phenotypic adaptation of *E. coli* to current clinical practices in both the human and veterinary setting. Patient vulnerability and antibiotic selective pressures provide an environment for the emergence of opportunistic MDR resistant *E. coli* based on the acquisition of at least two plasmid replicon groups, with numerous other horizontal DNA molecules also under selection. It will be of interest to evaluate the long-term stability of extra-chromosomal DNA in relation to antibiotic selective pressure and individual plasmid and resistance gene effects. In addition, it would also be of value to establish the reservoir potential of isolates as a source of resistance. It could be argued that the commensal background of the isolates requires a confluence of several factors for disease to occur, but should the horizontal transfer of plasmids to more pathogenic *E. coli* occur then the clinical significance of this increases exponentially.

## Transparency declarations

None to declare.

## Funding

This work was funded through a PhD case studentship jointly funded by the Biotechnology and Biological Sciences Research Council (R42122) and Zoetis (R82977).

## References

[bib0005] Altschul S.F., Madden T.L., Schaffer A.A., Zhang J.H., Zhang Z., Miller W., Lipman D.J. (1997). Gapped BLAST and PSI-BLAST: a new generation of protein database search programs. Nucleic Acids Res..

[bib0010] Baede V.O., Wagenaar J.A., Broens E.M., Duim B., Dohmen W., Nijsse R., Timmerman A.J., Hordijk J. (2015). Longitudinal study of extended-spectrum-β-lactamase- and ampC-producing enterobacteriaceae in household dogs. Antimicrob. Agents Chemother..

[bib0015] Bogaerts P., Huang T.-D., Bouchahrouf W., Bauraing C., Berhin C., El Garch F., Glupczynski Y. (2015). Characterization of ESBL- and ampC-producing enterobacteriaceae from diseased companion animals in europe. Microb. Drug Resist..

[bib0020] Bortolaia V., Larsen J., Damborg P., Guardabassi L. (2011). Potential pathogenicity and host range of extended-spectrum beta-lactamase-producing *Escherichia coli* isolates from healthy poultry. Appl. Environ. Microbiol..

[bib0025] Bortolaia V., Hansen K.H., Nielsen C.A., Fritsche T.R., Guardabassi L. (2014). High diversity of plasmids harbouring blaCMY-2 among clinical *Escherichia coli* isolates from humans and companion animals in the upper Midwestern USA. J. Antimicrob. Chemother..

[bib0030] Cao G., Allard M.W., Hoffman M., Monday S.R., Muruvanda T., Luo Y., Payne J., Rump L., Meng K., Zhao S., McDermott P.F., Brown E.W., Meng J. (2015). Complete sequences of six IncA/C plasmids of multidrug-resistant salmonella enterica subsp. enterica serotype newport. Genome Announc..

[bib0035] Chin C.-S., Alexander D.H., Marks P., Klammer A.A., Drake J., Heiner C., Clum A., Copeland A., Huddleston J., Eichler E.E., Turner S.W., Korlach J. (2013). Nonhybrid, finished microbial genome assemblies from long-read SMRT sequencing data. Nat. Methods.

[bib0040] Contreras-Moreira B., Vinuesa P. (2013). GET_HOMOLOGUES, a versatile software package for scalable and robust microbial pangenome analysis. Appl. Environ. Microbiol..

[bib0045] Croucher N.J., Page A.J., Connor T.R., Delaney A.J., Keane J.A., Bentley S.D., Parkhill J., Harris S.R. (2015). Rapid phylogenetic analysis of large samples of recombinant bacterial whole genome sequences using Gubbins. Nucleic Acids Res..

[bib0050] Darling A.E., Mau B., Perna N.T. (2010). ProgressiveMauve: multiple genome alignment with gene gain, loss and rearrangement. PLoS One.

[bib0055] Dashti A.A., Vali L., El-Shazly S., Jadaon M.M. (2014). The characterization and antibiotic resistance profiles of clinical *Escherichia coli* O25b-B2-ST131 isolates in Kuwait. BMC Microbiol..

[bib0060] Dierikx C.M., van Duijkeren E., Schoormans A.H.W., van Essen-Zandbergen A., Veldman K., Kant A., Huijsdens X.W., van der Zwaluw K., Wagenaar J.A., Mevius D.J. (2012). Occurrence and characteristics of extended-spectrum-β-lactamase- and AmpC-producing clinical isolates derived from companion animals and horses. J. Antimicrob. Chemother..

[bib0065] Edgar R.C. (2004). MUSCLE: Multiple sequence alignment with high accuracy and high throughput. Nucleic Acids Res..

[bib0070] Gibson J.S., Cobbold R.N., Trott D.J. (2010). Characterization of multidrug-resistant *Escherichia coli* isolated from extraintestinal clinical infections in animals. J. Med. Microbiol..

[bib0075] Grant J.R., Arantes A.S., Stothard P. (2012). Comparing thousands of circular genomes using the CGView Comparison Tool. BMC Genomics.

[bib0080] Haenni M., Saras E., Métayer V., Médaille C., Madec J.Y. (2014). High prevalence of blaCTX-M-1/IncI1/ST3 and bla CMY-2/IncI1/ST2 plasmids in healthy urban dogs in France. Antimicrob. Agents Chemother..

[bib0085] Hansen K.H., Bortolaia V., Nielsen C.A., Nielsen J.B., Schonning K., Agerso Y., Guardabassi L. (2016). Host-specific patterns of genetic diversity among incI1-Igamma and IncK plasmids encoding CMY-2 beta-lactamase in escherichia coli isolates from humans poultry meat, poultry, and dogs in Denmark. Appl. Environ. Microbiol..

[bib0090] Harris P.N.A. (2015). Clinical management of infections caused by enterobacteriaceae that express extended-spectrum β-lactamase and AmpC enzymes. Semin. Respir. Crit. Care Med..

[bib0095] Jolley K.A., Maiden M.C.J. (2010). BIGSdb: scalable analysis of bacterial genome variation at the population level. BMC Bioinf..

[bib0100] Joshi N., Fass J. (2011). Sickle: A Sliding-Window, Adaptive, Quality-Based Trimming Tool for FastQ Files (Version 1.33).

[bib0105] Kanehisa M., Sato Y., Morishima K. (2016). BlastKOALA and GhostKOALA: KEGG tools for functional characterization of genome and metagenome sequences. J. Mol. Biol..

[bib0110] Kearse M., Moir R., Wilson A., Stones-Havas S., Cheung M., Sturrock S., Buxton S., Cooper A., Markowitz S., Duran C., Thierer T., Ashton B., Meintjes P., Drummond A. (2012). Geneious Basic: an integrated and extendable desktop software platform for the organization and analysis of sequence data. Bioinformatics.

[bib0115] Koren S., Harhay G.P., Smith T.P.L., Bono J.L., Harhay D.M., Mcvey S.D., Radune D., Bergman N.H., Phillippy A.M. (2013). Reducing assembly complexity of microbial genomes with single-molecule sequencing. Genome Biol..

[bib0120] Li H., Durbin R. (2009). Fast and accurate short read alignment with burrows-wheeler transform. Bioinformatics.

[bib0125] Luo H., Zhang C.-T., Gao F. (2014). Ori-Finder 2, an integrated tool to predict replication origins in the archaeal genomes. Front. Microbiol..

[bib0130] Marshall B.M., Levy S.B. (2011). Food animals and antimicrobials: impacts on human health. Clin. Microbiol. Rev..

[bib0135] Murphy C.P., Reid-Smith R.J., Boerlin P., Weese J.S., Prescott J.F., Janecko N., Hassard L., McEwen S.A. (2010). *Escherichia coli* and selected veterinary and zoonotic pathogens isolated from environmental sites in companion animal veterinary hospitals in southern Ontario. Can. Vet. Journal-Revue Vet. Can..

[bib0140] Nakai H., Hagihara M., Kato H., Hirai J., Nishiyama N., Koizumi Y., Sakanashi D., Suematsu H., Yamagishi Y., Mikamo H. (2016). Prevalence and risk factors of infections caused by extended-spectrum β-lactamase (ESBL)-producing Enterobacteriaceae. J. Infect. Chemother..

[bib0145] Okubo T., Sato T., Yokota S., Usui M., Tamura Y. (2014). Comparison of broad-spectrum cephalosporin-resistant Escherichia coli isolated from dogs and humans in Hokkaido, Japan. J. Infect. Chemother..

[bib0150] Pitout J.D.D. (2010). Infections with extended-spectrum beta-lactamase-producing enterobacteriaceae: changing epidemiology and drug treatment choices. Drugs.

[bib0155] Rocha-Gracia R.C., Cortés-Cortés G., Lozano-Zarain P., Bello F., Martínez-Laguna Y., Torres C. (2015). Faecal Escherichia coli isolates from healthy dogs harbour CTX-M-15 and CMY-2 β-lactamases. Vet. J..

[bib0160] Sidjabat H.E., Seah K.Y., Coleman L., Sartor A., Derrington P., Heney C., Faoagali J., Nimmo G.R., Paterson D.L. (2014). Expansive spread of IncI1 plasmids carrying blaCMY-2 amongst Escherichia coli. Int. J. Antimicrob. Agents.

[bib0165] Stamatakis A. (2014). RAxML version 8: a tool for phylogenetic analysis and post-analysis of large phylogenies. Bioinformatics.

[bib0170] Stewart A.C., Osborne B., Read T.D. (2009). DIYA: a bacterial annotation pipeline for any genomics lab. Bioinformatics.

[bib0175] Suyama M., Torrents D., Bork P. (2006). PAL2NAL: robust conversion of protein sequence alignments into the corresponding codon alignments. Nucleic Acids Res..

[bib0180] Tamang M.D., Nam H.-M., Jang G.-C., Kim S.-R., Chae M.H., Jung S.-C., Byun J.-W., Park Y.H., Lim S.-K. (2012). Molecular characterization of extended-spectrum-β-lactamase-producing and plasmid-mediated AmpC β-lactamase-producing *Escherichia coli* isolated from stray dogs in South Korea. Antimicrob. Agents Chemother..

[bib0185] Villa L., García-Fernández A., Fortini D., Carattoli A. (2010). Replicon sequence typing of IncF plasmids carrying virulence and resistance determinants. J. Antimicrob. Chemother..

[bib0190] Wagner S., Gally D.L., Argyle S.A. (2014). Multidrug-resistant *Escherichia coli* from canine urinary tract infections tend to have commensal phylotypes, lower prevalence of virulence determinants and ampC-replicons. Vet. Microbiol..

[bib0195] Zankari E., Hasman H., Cosentino S., Vestergaard M., Rasmussen S., Lund O., Aarestrup F.M., Larsen M.V. (2012). Identification of acquired antimicrobial resistance genes. J. Antimicrob. Chemother..

[bib0200] Zerbino D.R., Birney E. (2008). Velvet: algorithms for de novo short read assembly using de Bruijn graphs. Genome Res..

[bib0205] Zerbino, D.R., 2010. velvet/contrib/shuffleSequences_fasta/shuffleSequences_fasta.pl.

