# Supplementary material for: Convergence of plasmid architectures drives emergence of multi-drug resistance in a clonally diverse Escherichia coli population from a veterinary clinical care setting
Source: Vet Microbiol. 2017 Nov;211:6–14. doi: 10.1016/j.vetmic.2017.09.016 (PMC5680696; doi:10.1016/j.vetmic.2017.09.016)
Supplement: Supplementary file 1 [file mmc1.docx]

| Isolate | Isolation date month/year | Resistance pattern | | | | | | | | AmpC Pheno | pAmpC PCR | Phylotype | FAB formula (*in silico* pMLST) | Replicon type (PCR derived) |
| --- | --- | --- | --- | --- | --- | --- | --- | --- | --- | --- | --- | --- | --- | --- |
| R1 (1283)* | 9/2006 | AMC | CEF | COT | CIP | | TET | |  | + | + | A | F31:A-:B- | FII I1 |
| R2 (127)* | 1/2008 | AMC | CEF | COT |  | | TET | |  | + | + | n/t | F:22:A6:B69 | FII I1 |
| R3 (1223)* | 9/2010 | AMC | CEF | COT | CIP | | TET | |  | + | + | A | F18:A-:B- | FII I1 |
| R4 (1428)* | 10/2010 | AMC | CEF |  |  | | TET | |  | + | + | D | F19:A-:B27  F4:A-:B- | FII I1 |
| R5 (746)* | 3/2010 | AMC | CEF |  |  | | TET | |  | + | + | D | F2:A-:B- | FII I1 |
| R6 (144)* | 2/2011 | AMC | CEF | COT |  | | TET | |  | + | + | D | F2:A-:B1 | FII I1 |
| R7 (1943)* | 12/2007 | AMC | CEF | COT | CIP | | TET | |  | + | + | B1 | F-:A-:B1 | B/O I1 |
| R8 (1176) | 9/2011 | AMC | CEF | COT | CIP | | TET | | GEN | + | - | B1 | F18:A-:B1 | FII FIB |
| R9 (1201) | 9/2011 | AMC | CEF | COT |  | | TET | |  | + | + | B1 |  | I1 |
| R10 (317)* | 3/2002 | AMC | CEF | COT | CIP | | TET | |  | + | + | B2 | F43:A-:B- | FII FIA I1 |
| R11a (136) | 2/2010 | AMC | CEF | COT |  | | TET | |  | + | - | D |  | - |
| R11b (1088) | 8/2010 | AMC | CEF |  |  | | TET | |  | + | - | D |  | - |
| R12 (1049) | 7/2006 | AMC | CEF | COT |  | | TET | | GEN | - | n/d | D |  | I2 |
| R13 (1258) | 8/2009 | AMC |  | COT | CIP | | TET | |  | - | n/d | A | F67:A6:B38 | FII FIA |
| R14 (1271) | 8/2008 | AMC |  | COT | CIP | | TET | |  | - | n/d | A |  | - |
| R15 (585) | 4/2009 | AMC | CEF | COT | CIP | TET | |  | | - | n/d | A |  | - |
| R16a (565) | 4/2011 | AMC | CEF |  | CIP | TET | | GEN | | - | n/d | B1 | F18:A-:B1 | FII B/O |
| R16b (737) | 5/2011 | AMC |  | COT | CIP | TET | | GEN | | - | n/d | B2 | F18:A-:B1 | FII FIB |

**Table S1**. Summary of resistant isolates used in this study allowing correlation of isolates used in previous study (Wagner et al. 2014) with the number designation referred to in the current study. Symbol * designates those isolates sequenced by SMRT. Resistance pattern identifies antimicrobials to which organisms were resistant: AMC = Amoxicillin clavulanate; CEF = Cephalexin; COT = co-trimoxazole; CIP = ciprofloxacin; TET = tetracycline; GEN = gentamicin. AmpC Pheno identifies which isolates were positive or negative for AmpC on the 4 disc phenotypic test. AmpC PCR identifies those isolates where plasmid associated AmpC genes could be identified by PCR (n/d designates not done).

| Isolate | Isolation date | Phylotype | FAB formula  (*in silico* pMLST) | PCR derived Replicon type |
| --- | --- | --- | --- | --- |
| S1 (162) | 1/2003 | B2 |  | - |
| S2 (1365) | 10/2011 | B2 |  | - |
| S3 (1568) | 11/2001 | B2 |  | - |
| S4 (1660) | 11/2001 | B2 |  | - |
| S5 (1711) | 11/2001 | B2 |  | - |
| S6 (073) | 1/2001 | B1 |  | X1 |
| S7 (1290) | 10/2002 | B2 |  | I2 R |
| S8 (1389) | 10/2001 | D | F43:A-:B- | FIA |
| S9 (78) | 1/2003 | B2 | - | FIB |
| S10 (1766) | 12/2001 | B2 | F-:A-:B59 | FII |
| S11 (1489) | 12/2000 | B2 | F29:A-:B10 | FII FIB |
| S12 (1333) | 10/2011 | D | F43:A-:B- | FII FIB |
| S14 (1105) | 8/2001 | B2 | - | FII I1 |
| S15 (1190)* | 9/2001 | B1 |  | B/O I1 |

**Table S2**. Summary of susceptible isolates used in this study allowing correlation of isolates used in previous study (Wagner et al. 2014) with the number designation referred to in the current study. Symbol * designates those isolates sequenced by SMRT. The FAB formula has been included except for those sequences where there was only a partial match with the pMLST database.

**Figure S1. Alignment and maximum-likelihood phylogenetic analysis of core IncI1 plasmid sequences.** IncI replicon PacBio sequences were used to identify other putative IncI1 plasmids from the NCBI nucleotide database. The sequences have been annotated with the predicted plasmid multi-locus sequence typing clonal group. The bacterial and animal hosts, from which the plasmid sequences were extracted, have also been annotated where available from submitted sequence metadata. Isolates from this study are identified by (◼).


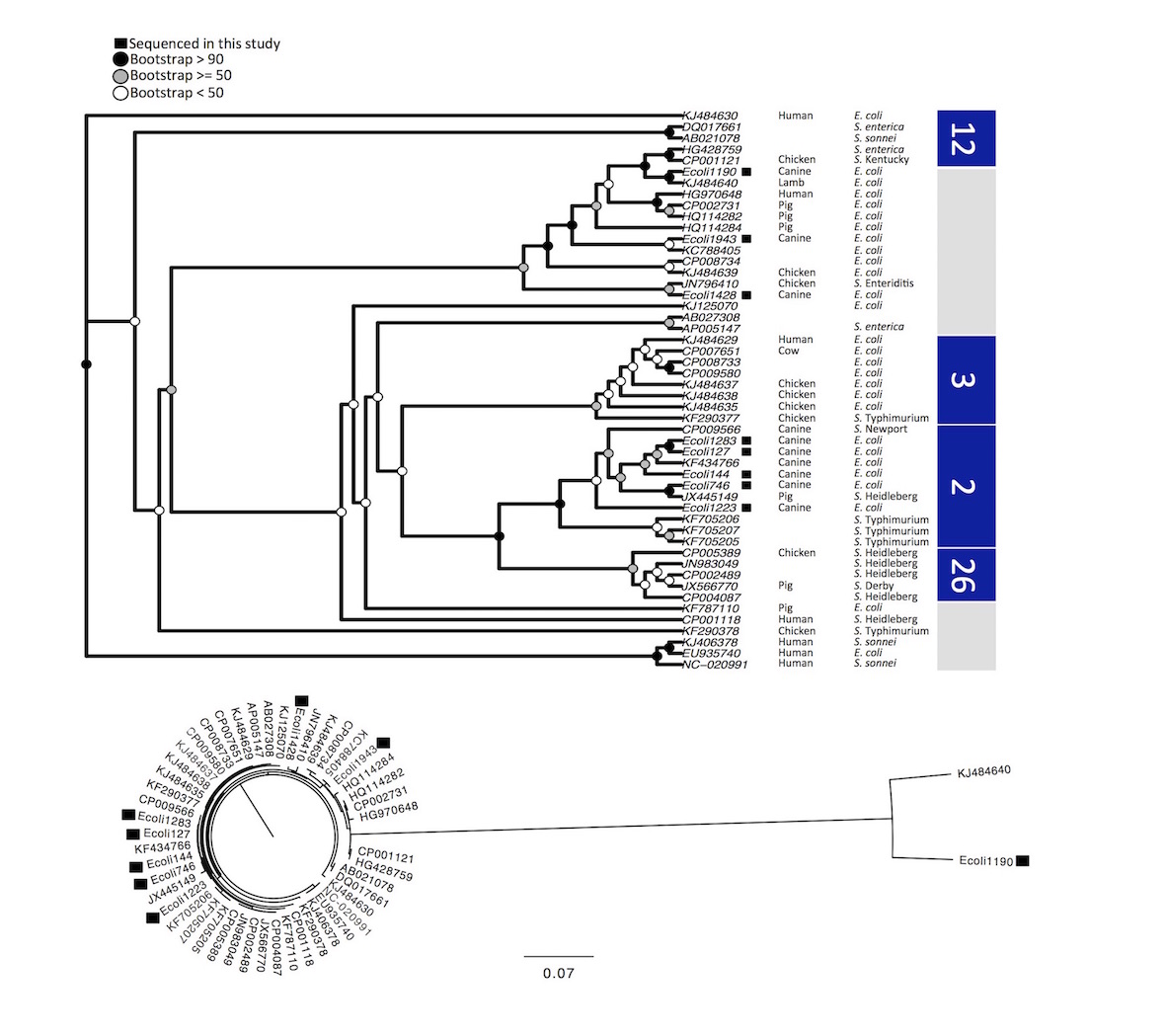


| **SUBID** | **BioProject** | **Biosample** | **Sample ID** | **Accession** | **Organism** |
| --- | --- | --- | --- | --- | --- |
| SUB2986438 | PRJNA402083 | SAMN07618129 | 746_chrom | CP023353 | E. coli 746 |
| SUB2986438 | PRJNA402083 | SAMN07618129 | 746_p62 | CP023354 | E. coli 746 |
| SUB2986438 | PRJNA402083 | SAMN07618129 | 746_p72 | CP023355 | E. coli 746 |
| SUB2986438 | PRJNA402083 | SAMN07618129 | 746_p95 | CP023356 | E. coli 746 |
| SUB2986438 | PRJNA402083 | SAMN07618128 | 317_chrom | CP023357 | E. coli 317 |
| SUB2986438 | PRJNA402083 | SAMN07618128 | 317_p100 | CP023358 | E. coli 317 |
| SUB2986438 | PRJNA402083 | SAMN07618127 | 1943_chrom | CP023359 | E. coli 1943 |
| SUB2986438 | PRJNA402083 | SAMN07618127 | 1943_p54 | CP023360 | E. coli 1943 |
| SUB2986438 | PRJNA402083 | SAMN07618127 | 1943_p80 | CP023361 | E. coli 1943 |
| SUB2986438 | PRJNA402083 | SAMN07618127 | 1943_p85 | CP023362 | E. coli 1943 |
| SUB2986438 | PRJNA402083 | SAMN07618126 | 144_p134 | CP023363 | E. coli 144 |
| SUB2986438 | PRJNA402083 | SAMN07618126 | 144_chrom | CP023364 | E. coli 144 |
| SUB2986438 | PRJNA402083 | SAMN07618126 | 144_p92 | CP023365 | E. coli 144 |
| SUB2986438 | PRJNA402083 | SAMN07618125 | 1428_chrom | CP023366 | E. coli 1428 |
| SUB2986438 | PRJNA402083 | SAMN07618125 | 1428_p111 | CP023367 | E. coli 1428 |
| SUB2986438 | PRJNA402083 | SAMN07618125 | 1428_p48 | CP023368 | E. coli 1428 |
| SUB2986438 | PRJNA402083 | SAMN07618125 | 1428_66 | CP023369 | E. coli 1428 |
| SUB2986438 | PRJNA402083 | SAMN07618125 | 1428_p96 | CP023370 | E. coli 1428 |
| SUB2986438 | PRJNA402083 | SAMN07618124 | 1283_chrom | CP023371 | E. coli 1283 |
| SUB2986438 | PRJNA402083 | SAMN07618124 | 1283_p109 | CP023372 | E. coli 1283 |
| SUB2986438 | PRJNA402083 | SAMN07618124 | 1283_p31 | CP023373 | E. coli 1283 |
| SUB2986438 | PRJNA402083 | SAMN07618124 | 1283_p3 | CP023374 | E. coli 1283 |
| SUB2986438 | PRJNA402083 | SAMN07618124 | 1283_p7 | CP023375 | E. coli 1283 |
| SUB2986438 | PRJNA402083 | SAMN07618124 | 1283_p92 | CP023376 | E. coli 1283 |
| SUB2986438 | PRJNA402083 | SAMN07618123 | 127_chrom | CP023377 | E. coli 127 |
| SUB2986438 | PRJNA402083 | SAMN07618123 | 127_p123 | CP023378 | E. coli 127 |
| SUB2986438 | PRJNA402083 | SAMN07618123 | 127_p39 | CP023379 | E. coli 127 |
| SUB2986438 | PRJNA402083 | SAMN07618123 | 127_p43 | CP023380 | E. coli 127 |
| SUB2986438 | PRJNA402083 | SAMN07618123 | 127_p91 | CP023381 | E. coli 127 |
| SUB2986438 | PRJNA402083 | SAMN07618123 | 127_p95 | CP023382 | E. coli 127 |
| SUB2986438 | PRJNA402083 | SAMN07618122 | 1223_chrom | CP023383 | E. coli 1223 |
| SUB2986438 | PRJNA402083 | SAMN07618122 | 1223_p147 | CP023384 | E. coli 1223 |
| SUB2986438 | PRJNA402083 | SAMN07618122 | 1223_p87 | CP023385 | E. coli 1223 |
| SUB2986438 | PRJNA402083 | SAMN07618121 | 1190_chrom | CP023386 | E. coli 1190 |
| SUB2986438 | PRJNA402083 | SAMN07618121 | 1190_p86 | CP023387 | E. coli 1190 |
| SUB2986438 | PRJNA402083 | SAMN07618120 | 1105_chrom | CP023388 | E. coli 1105 |
| SUB2986438 | PRJNA402083 | SAMN07618120 | 1105-p74 | CP023389 | E. coli 1105 |

**Table S3.** Bioproject identification and accession numbers for SMRT sequenced isolates in this study. _chrom designates chromosomal sequence and _p plasmid sequence. <https://www.ncbi.nlm.nih.gov/bioproject/?term=PRJNA402083>

| **Bioproject** | **Sample Identification** | **Accession number** | **Isolate** |
| --- | --- | --- | --- |
| PRJEB11950 | 746 | SAMEA3681619 | E. coli |
| PRJEB11950 | 1176 | SAMEA3681620 | E. coli |
| PRJEB11950 | 136 | SAMEA3681621 | E. coli |
| PRJEB11950 | 73 | SAMEA3681622 | E. coli |
| PRJEB11950 | 1428 | SAMEA3681623 | E. coli |
| PRJEB11950 | 1943 | SAMEA3681624 | E. coli |
| PRJEB11950 | 1271 | SAMEA3681625 | E. coli |
| PRJEB11950 | 1660 | SAMEA3681626 | E. coli |
| PRJEB11950 | 1088 | SAMEA3681627 | E. coli |
| PRJEB11950 | 1223 | SAMEA3681628 | E. coli |
| PRJEB11950 | 1568 | SAMEA3681629 | E. coli |
| PRJEB11950 | 162 | SAMEA3681631 | E. coli |
| PRJEB11950 | 1766 | SAMEA3681633 | E. coli |
| PRJEB11950 | 1290 | SAMEA3681634 | E. coli |
| PRJEB11950 | 1711 | SAMEA3681636 | E. coli |
| PRJEB11950 | 1389 | SAMEA3681637 | E. coli |
| PRJEB11950 | 1190 | SAMEA3681639 | E. coli |
| PRJEB11950 | 78 | SAMEA3681640 | E. coli |
| PRJEB11950 | 1489 | SAMEA3681641 | E. coli |
| PRJEB11950 | 1201 | SAMEA3681642 | E. coli |
| PRJEB11950 | 144 | SAMEA3681643 | E. coli |
| PRJEB11950 | 1105 | SAMEA3681644 | E. coli |
| PRJEB11950 | 317 | SAMEA3681645 | E. coli |
| PRJEB11950 | 565 | SAMEA3681646 | E. coli |
| PRJEB11950 | 1283 | SAMEA3681647 | E. coli |
| PRJEB11950 | 127 | SAMEA3681648 | E. coli |
| PRJEB11950 | 1365 | SAMEA3681649 | E. coli |
| PRJEB11950 | 737 | SAMEA3681650 | E. coli |
| PRJEB11950 | 585 | SAMEA3681651 | E. coli |
| PRJEB11950 | 1049 | SAMEA3681652 | E. coli |
| PRJEB11950 | 1333 | SAMEA3681653 | E. coli |
| PRJEB11950 | 1258 | SAMEA3681654 | E. coli |

**Table S4**. Illumina sequence data accession numbers for isolates used in this study. <http://www.ebi.ac.uk/ena/data/view/PRJEB11950>.
